# Prevalence of Hypertrophic Cardiomyopathy in the UK Biobank Population

**DOI:** 10.1001/jamacardio.2021.0689

**Published:** 2021-04-14

**Authors:** Luis R. Lopes, Nay Aung, Stefan van Duijvenboden, Patricia B. Munroe, Perry M. Elliott, Steffen E. Petersen

**Affiliations:** 1 Centre for Heart Muscle Disease, Institute of Cardiovascular Science, University College London, London, United Kingdom; 2 Barts Heart Centre, St Bartholomew’s Hospital, Barts Health NHS Trust, London, United Kingdom; 3 William Harvey Research Institute, NIHR Barts Biomedical Research Centre, Queen Mary University of London, Charterhouse Square, London, United Kingdom

## Abstract

This cohort study examines the prevalence of hypertrophic cardiomyopathy in the UK Biobank population.

Hypertrophic cardiomyopathy (HCM) causes considerable morbidity and mortality, including sudden death and death from heart failure and stroke.^1^ Hypertrophic cardiomyopathy is defined by unexplained left ventricular hypertrophy, which is defined as a maximum left ventricular wall thickness (MLVWT) of 15 mm or more (or ≥13 mm in relatives), out of proportion to loading conditions.^1^ The cited prevalence in the general population is 0.2%, based on studies in the community, in the military, and among athletes^2^ that relied on echocardiography. One study using cardiac magnetic resonance (CMR) reported a prevalence of 1.4% in the Multi-Ethnic Study of Atherosclerosis population.^3^

Limitations of these estimates included a narrow age range for some of the cohorts and exclusion of individuals with hypertension, regardless of the severity. The second of these is important, because hypertension is highly prevalent, including in up to 25% of patients with HCM carrying disease-causing variants.^4^ Relatives fulfilling criteria for familial HCM (with MLVWTs of 13-15 mm) were also not captured.

Cardiac magnetic resonance produces images with optimal myocardial (endocardial and epicardial) blood definition; mass and wall-thickness measurements are more accurate and reproducible compared with echocardiography, and milder hypertrophy in some segments (basal anterior or lateral or apical) can be missed by echocardiography.^1^ The use of CMR in family screening increases the yield of phenotype detection.

Machine learning facilitates rapid and accurate analysis of large CMR data sets.^5^ The UK Biobank is a population-based, prospective cohort study that enrolled 500 000 individuals aged 40 to 69 years (2006-2010).^6^ It contains information on demographics, health and lifestyle data, biological samples, and outcomes through linkages to electronic health records or registries; the imaging substudy commenced in 2014. Compared with the general population, the UK Biobank participants were more likely to be older, be female, live in less deprived areas, and have fewer comorbidities.^6^

## Methods

This study was covered by the general ethical approval for UK Biobank. The participants signed an electronic consent form at the time of their visit to the UK Biobank assessment center.

We adapted an automatic segmentation algorithm based on deep learning^5^ to measure LV wall thickness (maximum longitudinal distance between the epicardial and endocardial contours). We applied this method to calculate MLVWTs from short-axis measurements of 44 836 participants. Images with outlying MLVWT values and MLVWTs of 13 mm or more were manually validated by 2 European Association of Cardiovascular Imaging CMR level 3 experts (L.R.L. and N.A.). We excluded individuals with hypertension and aortic stenosis based on self-reported medical history and hospital episode statistics and those with phenocopies (Fabry disease, amyloidosis, glycogen storage diseases, and RASopathies). Data were collected from April 2014 to March 2020 and analyzed for this report from December 2020 to February 2021 with R version 3.6.1 (R Foundation for Statistical Computing).

## Results

After excluding individuals with hypertension (n = 14 970; including 607 with MLVWTs ≥13 mm), aortic stenosis (n = 80; 21 with MLVWTs ≥13 mm), and phenocopies (n = 40; 3 with MLVWT ≥13 mm; 0 with MLVWTs ≥15 mm), 29 826 individuals remained (mean [SD] age, 62.8 [7.7] years; 12 805 men [42.9%]). A total of 34 individuals had MLVWTs of 15 mm or more, for a prevalence of 0.11% (95% CI, 0.08%-0.15%); the prevalence was 0.22% (95% CI, 0.14%-0.30%) in male participants and 0.04% (95% CI, 0.01%-0.06%) in female participants. With a cutoff value of 13 mm or more, the prevalence was 0.96% (95% CI, 0.85%-1.07%) overall, 2.07% (95% CI, 1.82%-2.32%) in male participants, and 0.12% (95% CI, 0.07%-0.18%) in female participants. The Figure shows the prevalence for cutoff values of 13, 14, and 15 mm. Participants with MLVWT values of 13 to 14 mm vs those with values less than 13 mm are compared in the Table.

**Figure.**
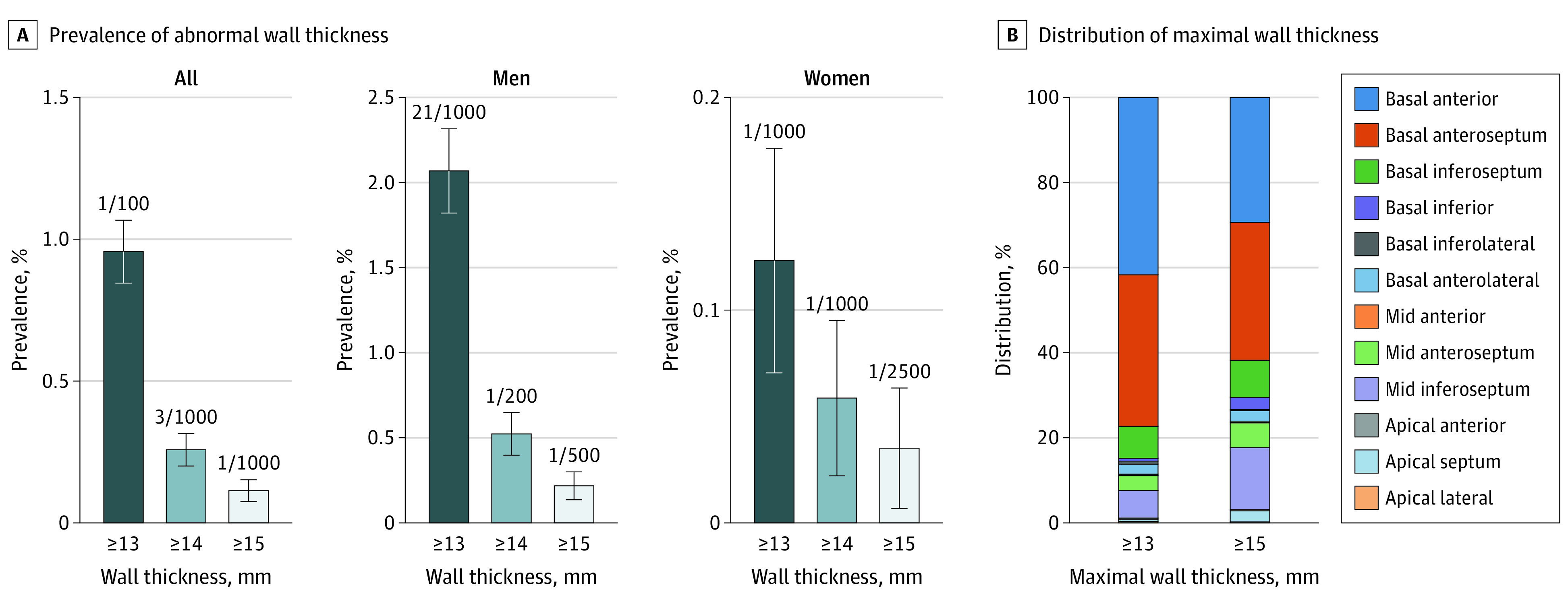
Prevalence of Left Ventricular Hypertrophy and Distribution of Maximal Wall Thickness The value above each bar corresponds to the proportion of individuals with maximal wall thickness greater than the threshold value.

**Table.** Characteristics of Participants in the UK Biobank

Characteristic	Maximal wall thickness, No. (%)			*P* value
	<13 mm	13-14 mm	≥15 mm	
No.	29 540	252	34	NA
Age, mean (SD), y	62.8 (7.7)	65.9 (8.3)	66.4 (7.4)	<.001
Male	12 540 (42.5)	237 (94.0)	28 (82.4)	<.001
Race/ethnicity				
White	28 620 (96.9)	244 (96.8)	33 (97.1)	.68
Asian	298 (1.0)	1 (0.4)	0	
Black	165 (0.6)	3 (1.2)	0	
Chinese	95 (0.3)	0	0	
Mixed	148 (0.5)	1 (0.4)	1 (2.9)	
Other	155 (0.5)	2 (0.8)	0	
Unknown	59 (0.2)	1 (0.4)	0	
BMI, mean (SD)	25.7 (4.0)	28.6 (4.2)	28.5 (5.1)	<.001
Degree-level education	20 222 (68.5)	175 (69.4)	20 (58.8)	.46
Smoking status				
Never	18 999 (64.9)	142 (57.3)	15 (45.5)	.001
Previous	9250 (31.6)	88 (35.5)	16 (48.5)	
Current	1024 (3.5)	18 (7.3)	2 (6.1)	
Regular alcohol intake	13 124 (44.7)	137 (54.8)	15 (45.5)	.006
Dyslipidemia	7084 (24.0)	80 (31.7)	9 (26.5)	.02
Diabetes	809 (2.7)	13 (5.2)	0	.04
Left ventricle, mean (SD)				
End-diastolic volume, mL	144.7 (32.5)	170.6 (42.2)	170.3 (36.9)	<.001
End-systolic volume, mL	59.0 (18.1)	72.0 (24.6)	72.1 (16.8)	<.001
Stroke volume, mL	85.7 (18.2)	98.6 (24.2)	98.2 (29.6)	<.001
Ejection fraction, %	59.6 (5.8)	58.2 (7.2)	57.2 (7.8)	<.001
Mass, g	82.0 (20.6)	124.7 (24.1)	142.9 (30.3)	<.001
Maximal wall thickness, mean (SD), mm	9.0 (1.4)	13.6 (0.5)	16.0 (1.0)	NA

Abbreviations: BMI, body mass index (calculated as weight in kilograms divided by height in meters squared); NA, not applicable.

Guidelines^1^ consider that MLVWT values of 15 mm or more in White individuals and 20 mm or more in Black individuals are in favor of HCM vs hypertensive heart disease. If we included these participants regardless of hypertension status, prevalence of HCM was 0.22% (95% CI, 0.18%-0.27%). The presence of MLVWT values of 15 mm or more was mostly located in the basal anterior and anteroseptal segments (Figure).

## Discussion

To our knowledge, this is the largest HCM prevalence study based on imaging. Our estimates of 0.11% to 0.22% are consistent with previous reports; the sex-based difference was present in previous studies to a similar degree^2,3^ and might be partially explained by wall thickness not being adjusted to sex or body size. It is not entirely clear what the causative mechanism of left ventricular hypertrophy for the cases within 13 to 14 mm is. Environmental factors might be a relevant contributor. The higher volumes might be partially explained by the same reason, associated with larger body sizes; left ventricular dimensions and derived volumes are known to be smaller in echocardiography compared with CMR, because the contrast between the blood pool and endocardial border is worse. Older ages may reflect the age-associated penetrance of HCM. There is an increased prevalence of women and healthy volunteer selection bias in the UK Biobank, which might limit generalizability of our findings to more diverse populations. However, the use of a more accurate imaging technique, the very large cohort (>40 000), the inclusion of individuals with MLVWT values of 13 mm or more, and a more nuanced approach regarding the presence of hypertension, likely compensated for these limitations.
